# Metagenomic next generation sequencing for the diagnosis of tuberculosis meningitis: A systematic review and meta-analysis

**DOI:** 10.1371/journal.pone.0243161

**Published:** 2020-12-01

**Authors:** Guocan Yu, Wuchen Zhao, Yanqin Shen, Pengfei Zhu, Hong Zheng

**Affiliations:** Zhejiang Tuberculosis Diagnosis and Treatment Center, Zhejiang Chinese Medicine and Western Medicine Integrated Hospital, Hangzhou, Zhejiang, China; The University of Georgia, UNITED STATES

## Abstract

**Background:**

Tuberculous meningitis (TBM) is a severe form of extrapulmonary tuberculosis and its early diagnosis is very difficult leading to present with severe disability or die. The current study aimed to assess the accuracy of metagenomic next generation sequencing (mNGS) for TBM, and to identify a new test for the early diagnosis of TBM.

**Methods:**

We searched for articles published in Embase, PubMed, Cochrane Library, China National Knowledge Infrastructure, and Wanfang Data up to June 30, 2020 for studies that assessed the efficacy of mNGS for the diagnosis of TBM. Then, the accuracy between mNGS and a composite reference standard (CRS) in these articles was compared using the meta-analysis approach.

**Results:**

Four independent studies with 342 samples comparing mNGS and a CRS were included in this study. The sensitivity of mNGS for TBM diagnosis ranged from 27% to 84%. The combined sensitivity of mNGS was 61%, and the I^2^ value was 92%. Moreover, the specificity of mNGS for TBM diagnosis ranged from 96% to 100%. The combined specificity of mNGS was 98%, and the I^2^ value was 74%. The heterogeneity between studies in terms of sensitivity and specificity was significant. The area under the curve (AUC) of the summary receiver operating characteristic curve (SROC) of mNGS for TBM was 0.98.

**Conclusions:**

The sensitivity of mNGS for TBM diagnosis was moderate. Furthermore, the specificity was extremely high, and the AUC of the SROC indicated a very good diagnostic efficacy. mNGS could be used as an early diagnostic method for TBM, however, the results should be treated with caution for the heterogeneity between studies was extremely significant.

**Systematic review registration:**

INPLASY202070100.

## Introduction

Tuberculosis (TB) is a serious threat to human health worldwide [1]. A high proportion of individuals are infected with tuberculosis annually, and some die from related diseases. In 2018, there were about 10 million new cases of TB worldwide and about 1.45 million deaths from TB [2]. Moreover, this condition is the leading cause of death from infectious diseases [2]. In addition to pulmonary tuberculosis (PTB), Mycobacterium tuberculosis (MTB) can also cause extrapulmonary tuberculosis (EPTB) [3]. Tuberculous meningitis (TBM) is a highly lethal type of EPTB. Although it accounts for a relatively small proportion (1%–5%) of new TB cases, 50% of individuals with TBM can die or present with severe disability [4]. Failure to facilitate early diagnosis and treatment is one of the primary causes for these serious complications [5]. Thus, early diagnosis can improve the prognosis of TBM. Conventional mycobacterium detection techniques do not meet the need for early detection [4]. Acid-fast bacilli (AFB) smear is the cheapest and most convenient method for the diagnosis of TB. However, its sensitivity in the cerebrospinal fluid (CSF) is extremely low (≤15%), especially in the absence of professional microscopists in the laboratory [6]. Mycobacterium culture in the CSF and AFB smear have extremely low sensitivity, and these methods are time-consuming. Therefore, culture results cannot be used as an indicator of early diagnosis [7]. Currently, in some cases, empirical antituberculous therapy is provided when TBM cannot be ruled out. Hence, the early and rapid diagnosis of TBM should be improved.

The advent of nucleic acid amplification tests (NAATs) has facilitated early and rapid TB diagnosis [8]. Xpert MTB/RIF (Cepheid, Sunnyvale, CA) is a representative NAAT that is extensively used for the diagnosis of TB [9]. Furthermore, according to the recommendation of the World Health Organization, it is used as the initial test for the diagnosis of TBM [10].

Recently, with the advancement of molecular diagnostic technology, metagenomic next generation sequencing (mNGS) technology was developed, and it can provide information on the genomic DNA sequences of microorganisms. The mNGS technology is a new detection method, which allows for an unbiased and detailed test of the total DNA or RNA content of all currently known pathogenic microorganisms [11]. Thus, it has been increasingly used [12]. mNGS could detect pathogenic microorganisms from a wide range of clinical specimens, including CSF [11, 13, 14]. Recent studies have shown that the sensitivity and specificity of mNGS for detecting pathogenic bacteria are significantly better than that of culture [15]. However, studies on the diagnostic efficacy of mNGS-based detection of MTB DNA for TBM were controversial [16, 17]. Moreover, relevant systematic evaluation and meta-analysis of the diagnostic efficacy of mNGS for TBM have not been conducted. Therefore, this study evaluated and compared the efficacy of mNGS for the diagnosis of TBM using CSF specimens versus a composite reference standard (CRS) to identify a new technique for the early diagnosis of TBM.

## Methods

### Design and registration

A systematic review and meta-analysis of the diagnostic accuracy of mNGS was conducted. The study protocol was registered on the International Platform of Registered Systematic Review and Meta-Analysis Protocols (INPLASY) (registration number: INPLASY202070100) [18]. This study was conducted in accordance with the Preferred Reporting Items for Systematic Reviews and Meta-Analyses statements [19]. Ethical approval was not required for the study.

### Information sources

We searched studies, which assessed the diagnostic accuracy of mNGS for TBM, in Embase, PubMed, Cochrane Library, China National Knowledge Infrastructure (CNKI), and Wanfang Database up to June 30, 2020. Moreover, the references cited in the reviews were evaluated for other possible studies.

### Search strategy

Guocan Yu and Wuchen Zhao conducted the search strategies. There were no language restrictions in our search process. Search strategy of PubMed was listed as follows:

#1 "Tuberculosis, Meningeal"[Mesh] OR “Meningeal Tuberculoses” OR “Meningeal Tuberculosis” OR “Tuberculoses, Meningeal” OR “TB Meningitis” OR “TB Meningitides” OR “Tubercular Meningitis” OR “Meningitides, Tubercular” OR “Meningitis, Tubercular” OR “Tubercular Meningitides” OR “Meningitis, Tuberculous” OR “Meningitides, Tuberculous” OR “Tuberculous Meningitides” OR “Tuberculous Meningitis” OR “Tuberculosis Meningitis” OR “Meningitides, Tuberculosis” OR “Meningitis, Tuberculosis” OR “Tuberculosis Meningitides” OR “Tuberculous Hypertrophic Pachymeningitis” OR “Hypertrophic Pachymeningitides, Tuberculous” OR “Hypertrophic Pachymeningitis, Tuberculous” OR “Pachymeningitides, Tuberculous Hypertrophic” OR “Pachymeningitis, Tuberculous Hypertrophic” OR “Tuberculous Hypertrophic Pachymeningitides”

#2 “Extrapulmonary tuberculosis” OR “Extra pulmonary tuberculosis”

#3 "Meningitis"[Mesh] OR Meningitides OR Pachymeningitis OR Pachymeningitides

#4 "Cerebrospinal Fluid"[Mesh] OR “Cerebrospinal Fluids” OR “Fluid, Cerebrospinal” OR “Fluids, Cerebrospinal” OR “Cerebro Spinal Fluid” OR “Cerebro Spinal Fluids” OR “Fluid, Cerebro Spinal” OR “Fluids, Cerebro Spinal” OR “Spinal Fluid, Cerebro” OR “Spinal Fluids, Cerebro”

#5 #1 OR #2 OR #3 OR #4

#6 “Metagenomic Next-Generation Sequencing” OR mNGS

#7 #5 AND #6

Similar search formulae were used for the Cochrane Library, Embase, CNKI, and Wanfang databases.

### Eligibility criteria

#### Type of study

We included different types of studies, such as case-control, retrospective, and prospective studies. Full text original studies that assessed the efficacy of mNGS for the diagnosis of TBM were included. The true-positive (TP), false-positive (FP), false-negative (FN), and true-negative (TN) values for the assay can be extracted or calculated directly from the studies. However, case reports, articles written in languages other than Chinese and English, studies with < 10 specimens, conference reports, and abstracts without full articles were excluded.

#### Patients

Studies with patients diagnosed with TBM via mNGS were included, and there were no restrictions on gender, age, and nations.

#### Index tests

mNGS was considered as the index test.

#### Main outcomes

Sensitivity and the specificity of mNGS were considered as the main outcome.

#### Reference standards

CRS was defined as the reference standard in this study. The reference standards in CRS comprised clinical symptoms, radiographic features, immunological index, biochemical test results, smears, culture, histopathology, and response to anti-tuberculosis drugs. Patients with some or all factors who had positive results were considered positive for TBM. Meanwhile, cases were considered as non-TBP if all the test results were negative.

### Literature screening and selection

The primary search records were imported into the ENDNOTE X9.2 literature management software, according to the eligibility criteria. Two investigators (Guocan Yu and Wuchen Zhao) independently assessed the candidate articles by reviewing their titles and abstracts, followed by the full text, for inclusion. Discrepancies between the two investigators was resolved via a discussion with a third investigator (Hong Zheng).

### Data extraction

We extracted data including those on first author name; publication year; country where the study was conducted; TP, FP, FN, and TN values for the assay; type of research; patient selection method; sample pre-treatment method; homogenization; and sample condition along with other parameters. The same researchers independently extracted relevant data from the included articles; the data obtained were then cross-checked. Discrepancies between the two information sets were settled via a discussion with a third investigator, similar to the method used during the literature selection phase.

### Quality evaluation

Based on the reference standards, to independently assess study quality, the two investigators independently used a revised tool for the Quality Assessment of Diagnostic Accuracy Studies (QUADAS-2) [20]. The discrepancy between reviewers was resolved via a discussion with a third investigator (Hong Zheng). The Funnel chart was used to evaluate whether publication bias existed in the included studies.

### Data synthesis and statistical analysis

We first obtained the TP, FP, FN, and TN values in each study. Next, using the bivariate random-effects models, the combined sensitivity and specificity with 95% confidence interval (CI) between mNGS and CRS were calculated and compared. The forest plots for the sensitivity and specificity of each study were generated. The areas under the curve (AUC) of the summary receiver operating characteristic (SROC) were subsequently calculated. *I*^2^ statistics was used to assess heterogeneity between the studies and the reference standard. A value of 0% indicated the absence of heterogeneity and a value > 50%, substantial heterogeneity [21]. Via meta-regression and subgroup analyses, we assessed the following: different types of studies, patient selection method, sample pre-treatment method, sample conditions, and homogenization as potential sources of heterogeneity. At least four published studies were required to perform meta-analysis for predefined variable types. Stata version 15.0 (Stata Corp., College Station, TX, the USA) with *midas* module were used to generate the forest plots for the sensitivity and specificity with 95% CI in each study and to carry out meta-analyses and meta-regression analyses. *midas* is a command for idiot-proof implementation of the contemporary statistical methods for meta-analysis of diagnostic test accuracy [22].

## Results

### Characteristics of the studies

**Fig 1:**
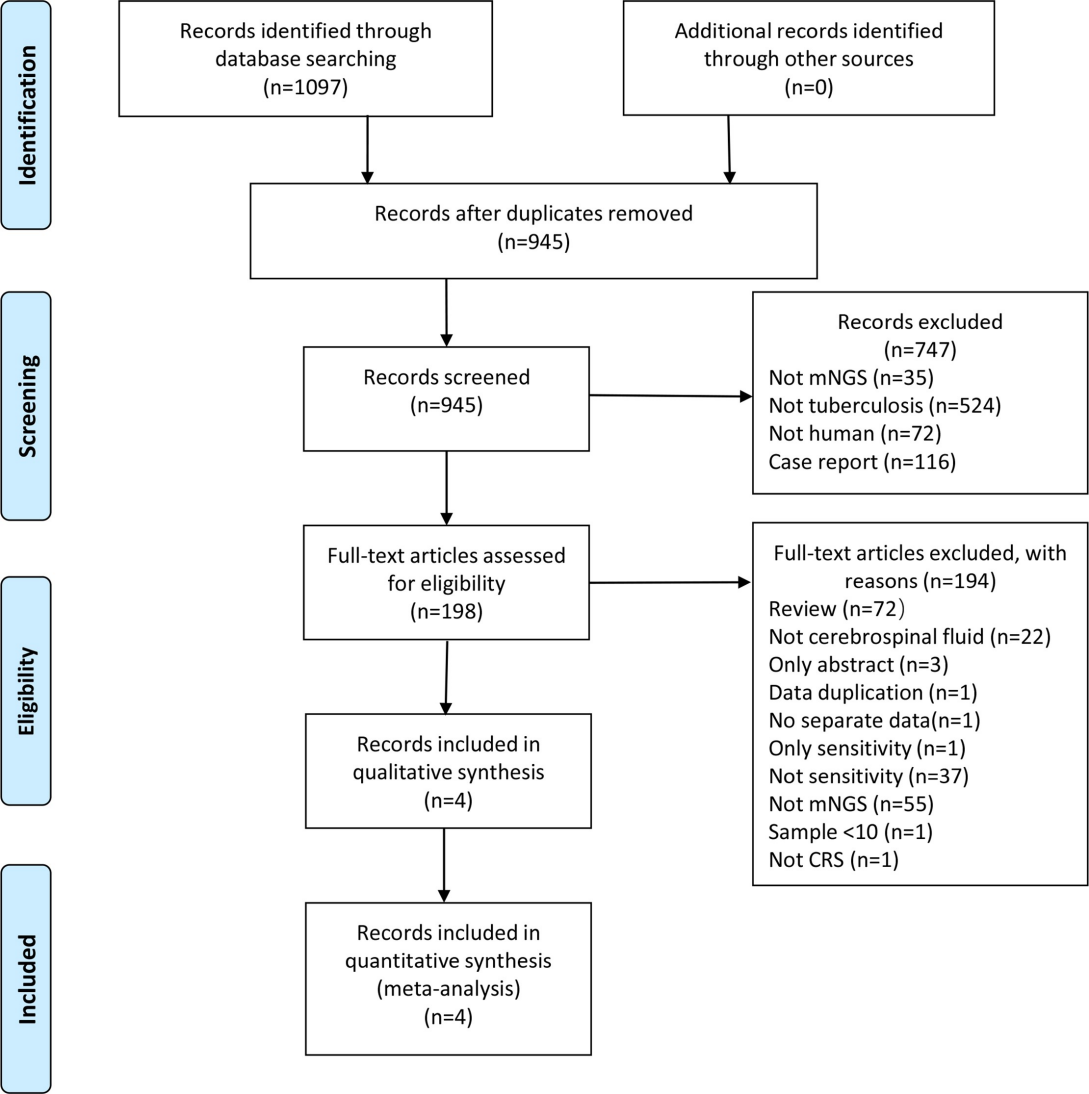
Flow chart of literature retrieval. In total, 507, 53, 0, 242, and 295 articles were found in Embase, PubMed, Cochrane Library, CNKI, and Wanfang Data, respectively.

After searching relevant databases using our search strategy, we identified 1097 candidate articles. Of these articles, four, including two retrospective and two prospective, studies met the inclusion criteria [15–17, 23] (Fig 1). The kappa index of agreement for the selection of studies and data extraction was 0.665 (95% confidence interval [CI]: 0.355–0.975) between the two investigators. All studies were conducted in low-income areas with a high prevalence of TB. All articles have been published in English. CSF was used in all studies. The size of the specimen ranged from 29 to 213, with a median specimen size of 50 and a total specimen size of 342. All characteristics of the included study are depicted in Table 1. We excluded one article that reported sensitivity, but not specificity [24], and another one that presented data that were discussed in an article we already included [25].

**Table 1:** Characteristics of the included studies.

Author	Year	County	sample type	reference	N	TP	FP	FN	TN	Type of research	Sample condition	Homogenisation	sample pre-treatment	patient selection method	patient population
Wang S	2019	China	CSF	CRS	29	18	0	5	6	Retrospective	Frozen	Mechanical	With bead-beating	Convenience	Low
Zhou X	2019	China	CSF	CRS	49	7	1	9	32	Prospective	Fresh/Frozen	No	Without bead-beating	Consecutive	Low
Xing X	2020	China	CSF	CRS	213	12	6	32	163	Prospective	Frozen	No	With bead-beating	Convenience	Low
Yan L	2020	China	CSF	CRS	51	38	0	7	6	Retrospective	Fresh	Mechanical	With bead-beating	Convenience	Low

CSF, cerebrospinal fluid; CRS, composite reference standard; TP, true-positive; FP, false-positive; FN, false-negative; TN, true-negative.

### Study quality

**Fig 2:**
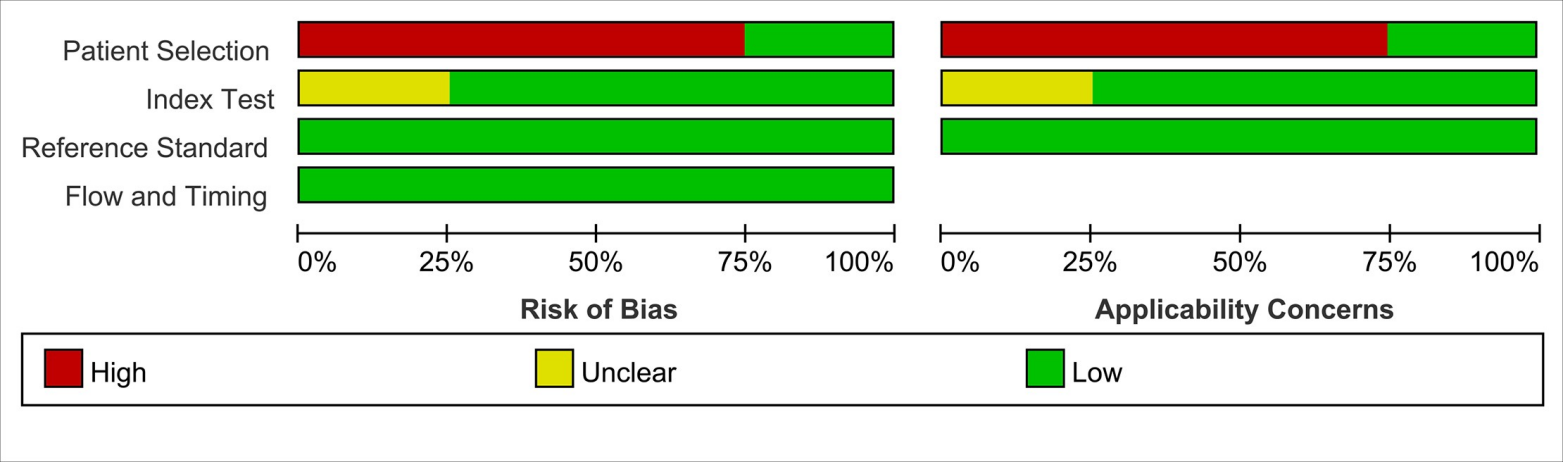
Methodological quality graphs (risk of bias and applicability concerns) were presented as percentages across the included studies using a composite reference standard.

The overall methodological quality of the included studies, using a CRS as the gold reference, is presented in Fig 2. The risk of bias was primarily attributed to patient selection and index test. The risk of bias from the flow and timing was low, as was the risk of bias from the reference standard. An assessment of publication bias was not performed due to the limited number of studies included (<10).

### Diagnostic accuracy of mNGS for TBM

**Fig 3:**
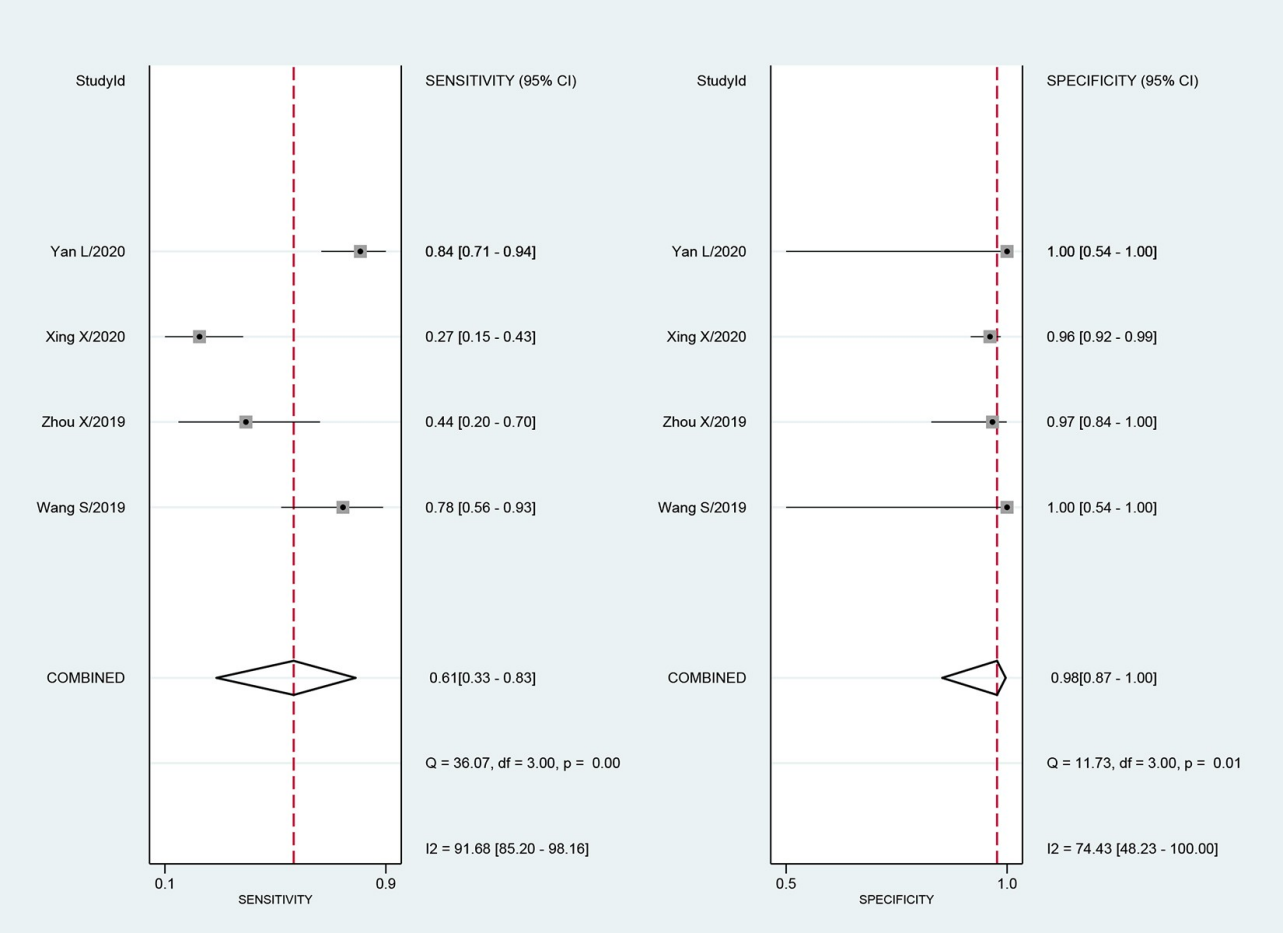
Forest plot for the sensitivity and specificity of mNGS for the diagnosis of tuberculosis meningitis using a composite reference standard.

Four studies included 342 samples with a CRS. The sensitivity of mNGS for TBM ranged from 27% (95% CI: 15–43) to 84% (95% CI: 71–94). The combined sensitivity of mNGS for the diagnosis of TBM was 61% (95% CI: 33–83), and the I^2^ value was 92% (95% CI: 85–98). The specificity of mNGS ranged from 96% (95% CI: 92–99) to 100% (95% CI: 54–100). Furthermore, the combined specificity of mNGS was 98% (95% CI: 87–100), and the I^2^ value was 74% (95% CI: 48–100) (Fig 3). The heterogeneity between studies in terms of sensitivity and specificity was significant. The AUC of the SROC was 0.98 (95% CI: 0.96–0.99).

We explored the heterogeneity among studies using subgroup, meta-regression, and sensitivity analyses. Subgroup and meta-regression analyses were performed on the predefined subgroups for the different types of studies, patient selection method, sample pre-treatment method, and homogenization used in the assay. Meta-regression analysis demonstrated that the different types of studies (prospective vs. retrospective) had effect on the sensitivity and specificity of mNGS for TBM compared with CRS (32% vs. 82%, meta-regression *P* < 0.01; 97% vs. 100%, meta-regression *P* < 0.01). The same results were also observed in studies using different homogenization methods (mechanical or not) (Table 2). With or without bead-beating for sample pre-treatment might have an effect on the specificity of mNGS (98% vs. 97%, meta-regression *P* = 0.03), but not sensitivity (66% vs. 43%, meta-regression *P* = 0.43). The patient selection methods (consecutive or convenience) had no effect on the sensitivity and specificity of mNGS (meta-regression *P* > 0.05). However, limited data from relevant studies were included to facilitate subgroup and sensitivity analysis. One study was unable to distinguish sample conditions [23], so meta-regression analysis for sample conditions could not be performed.

**Table 2:** Meta-regression analysis for different parameters.

Parameter		Sensitivity (95% CI)	Specificity (95% CI)
Type of study	Prospective (2 studies)	32% (20–43%)	97% (94–99%)
	Retrospective (2 studies)	82% (73–91%)	100% (100–100%)
	Meta-regression *P-*value	<0.01	<0.01
Homogenization	Mechanical (2 studies)	82% (73–91%)	100% (100–100%)
	No (2 studies)	32% (20–43%)	97% (94–99%)
	Meta-regression *P-*vaule	<0.01	<0.01
Sample pre-treatment	With bead-beating (3 studies)	66% (38–93%)	98% (94–100%)
	Without bead-beating (1 study)	43% (11–98%)	97% (91–100%)
	Meta-regression *P-*vaule	0.39	0.03
Patient selection	Consecutive (1 study)	43% (11–98%)	97% (91–100%)
	Convenience (3 studies)	66% (38–93%)	98% (94–100%)
	Meta-regression *P-*vaule	0.44	0.21

CI: confidence interval

## Discussion

Similar to other types of EPTB, the paucibacillary nature makes early diagnosis of TBM more difficult [26]. Delays in the diagnosis and treatment of TBM can lead to serious complications such as severe disability or death. This can significantly affect the patient’s prognosis and quality of life [27]. Lumbar puncture is a vital step in the diagnosis of patients. CSF obtained via lumbar puncture is the most common specimen used to diagnose TBM. Due to the low bacterial content in the sample, TBM is still challenging to diagnose via AFB and CSF culture [28]. By contrast, TBM is easily confused with cryptococcal meningitis or viral meningitis, and the diagnosis of the causative organism is quite difficult [16]. Hence, TBM is often missed or misdiagnosed. Therefore, to reduce mortality and disability from TBM and to facilitate early identification of the causative organisms, rapid and effective diagnostic methods should be developed to obtain a differential diagnosis of TBM.

NAATs, such as Xpert MTB/RIF assay and the loop-mediated isothermal amplification assay, are fast and efficient detection methods. Hence, they have been widely used in the early diagnosis of TB [29, 30]. Moreover, they have an extremely good diagnostic performance in paucibacillary EPTB [31]. However, these methods have limitations. That is, a single test can only detect MTB DNA and can only provide indirect assistance in the differential diagnosis of TBM.

The mNGS technology is a new detection method for all currently known pathogenic microorganisms, including MTB [12]. mNGS allows for an unbiased and detailed test of the total DNA or RNA content of the microbiome [32]. Moreover, it takes about 3 days to obtain the results, which is still relatively fast compared with culture. mNGS can detect a wide range of pathogenic microorganisms, and it is useful in obtaining the differential diagnosis of suspected TBM. The current findings on the use of mNGS for the diagnosis of TBM are controversial. Moreover, systematic review and meta-analysis of the use of mNGS for the diagnosis of TBM were not conducted. Therefore, the current study was conducted.

The current study included four studies that conducted a comparison between mNGS and CRS, showing that the combined sensitivity and specificity of mNGS for the diagnosis of TBM were 61% (95% CI: 33–83) and 98% (95% CI: 87–100), respectively. The sensitivity widely varied among the studies. The AUC of the SROC was 0.98 (95% CI: 0.96–0.99), thereby indicating that mNGS had an extremely good diagnostic performance for TBM. However, a substantial level of heterogeneity was observed, regardless of sensitivity and specificity. Therefore, the results should be treated with caution. Publication bias was not assessed because of the limited number of studies included. The type of research, patient selection method, sample pre-treatment method, sample conditions, and homogenization differed among the studies. The patient selection methods were non-consecutive for most of the included studies. However, most of the studies used bead-beating for sample pre-treatment. Meta-regression analysis demonstrated that the different types of studies and homogenization methods had effect on the sensitivity and specificity of mNGS for TBM compared with CRS. Sample pre-treatment methods might have effect on the specificity of mNGS, but not sensitivity. Different homogenization and sample pre-treatment methods might lead to different distribution of MTB in solution, which might affect the diagnostic performance of mNGS. However, whether these methods really affect the diagnostic performance of mNGS remains to be explored further, and the mechanisms need to be clarified further. The patient selection methods had no effect on the sensitivity and specificity of mNGS. However, the limited number of studies included did not allow for further subgroup. These factors might be a source of heterogeneity among studies. The impact of these factors on diagnostic validity needs to be further confirmed by larger clinical trials. Furthermore, sensitivity analysis could not be conducted due to the limited number of studies. For paucibacillary TBM, culture might not be a perfect reference standard. However, CRS, which uses a combination of factors, might be a more appropriate reference standard. In addition, the number of studies comparing the diagnostic efficacy between culture and mNGS for TBM is extremely limited. Therefore, in this study, CRS was used as the reference standard. The CRS used in the included studies was also inconsistent. For example, some studies had not included response to treatment, which might be a source of heterogeneity.

This meta-analysis had several limitations. Despite a comprehensive search, some articles might have been missed, and the number of studies included in this meta-analysis is limited. In addition, some studies cannot distinguish meningitis data, which might have led to some bias in the results. In addition, studies on the diagnosis of other pathogens via mNGS were not included in the current analysis. In addition, the CRS differed among the studies. Different CRS might lead to changes in patient classification and thus affect the sensitivity and specificity of the test. The heterogeneity among the studies was substantial, and the combined estimates must be treated with caution.

## Conclusions

This study showed that the sensitivity of mNGS for the diagnosis of TBM was moderate. Moreover, the specificity was extremely high, and the AUC indicated a very good diagnostic efficacy. mNGS could be used as an early diagnostic method for TBM. However, the heterogeneity between studies was extremely significant. Hence, the results should be treated with caution.

## Supporting information

S1 Checklist PRISMA 2009 checklist. (DOC)

S1 File Data analyzed in this study, including search strategy. (DOCX)
