# Supplementary material for: Metagenomic next generation sequencing for the diagnosis of tuberculosis meningitis: A systematic review and meta-analysis
Source: PLoS One. 2020 Dec 1;15(12):e0243161. doi: 10.1371/journal.pone.0243161 (PMC7707562; doi:10.1371/journal.pone.0243161)
Supplement: S1 File — (DOCX) [file pone.0243161.s002.docx]

Pubmed and Cochrane

#1 "Tuberculosis, Meningeal"[Mesh] OR “Meningeal Tuberculoses” OR “Meningeal Tuberculosis” OR “Tuberculoses, Meningeal” OR “TB Meningitis” OR “TB Meningitides” OR “Tubercular Meningitis” OR “Meningitides, Tubercular” OR “Meningitis, Tubercular” OR “Tubercular Meningitides” OR “Meningitis, Tuberculous” OR “Meningitides, Tuberculous” OR “Tuberculous Meningitides” OR “Tuberculous Meningitis” OR “Tuberculosis Meningitis” OR “Meningitides, Tuberculosis” OR “Meningitis, Tuberculosis” OR “Tuberculosis Meningitides” OR “Tuberculous Hypertrophic Pachymeningitis” OR “Hypertrophic Pachymeningitides, Tuberculous” OR “Hypertrophic Pachymeningitis, Tuberculous” OR “Pachymeningitides, Tuberculous Hypertrophic” OR “Pachymeningitis, Tuberculous Hypertrophic” OR “Tuberculous Hypertrophic Pachymeningitides”

#2 “Extrapulmonary tuberculosis” OR “Extra pulmonary tuberculosis”

#3 "Meningitis"[Mesh] OR Meningitides OR Pachymeningitis OR Pachymeningitides

#4 "Cerebrospinal Fluid"[Mesh] OR “Cerebrospinal Fluids” OR “Fluid, Cerebrospinal” OR “Fluids, Cerebrospinal” OR “Cerebro Spinal Fluid” OR “Cerebro Spinal Fluids” OR “Fluid, Cerebro Spinal” OR “Fluids, Cerebro Spinal” OR “Spinal Fluid, Cerebro” OR “Spinal Fluids, Cerebro”

#5 #1 OR #2 OR #3 OR #4

#6 “Metagenomic Next-Generation Sequencing” OR mNGS

#7 #5 AND #6

EMBASE

#1 'tuberculous meningitis'/exp OR 'Meningeal Tuberculoses'OR 'Meningeal Tuberculosis'OR 'Tuberculoses, Meningeal' OR 'TB Meningitis' OR 'TB Meningitides' OR 'Tubercular Meningitis' OR 'Meningitides, Tubercular' OR ‘Meningitis, Tubercular’ OR ‘Tubercular Meningitides’ OR ‘Meningitis, Tuberculous’ OR ‘Meningitides, Tuberculous’ OR ‘Tuberculous Meningitides’ OR ‘Tuberculous Meningitis’ OR ‘Tuberculosis Meningitis’ OR ‘Meningitides, Tuberculosis’ OR ‘Meningitis, Tuberculosis’ OR ‘Tuberculosis Meningitides’ OR ‘Tuberculous Hypertrophic Pachymeningitis’ OR ‘Hypertrophic Pachymeningitides, Tuberculous’ OR ‘Hypertrophic Pachymeningitis, Tuberculous’ OR ‘Pachymeningitides, Tuberculous Hypertrophic’ OR ‘Pachymeningitis, Tuberculous Hypertrophic’ OR ‘Tuberculous Hypertrophic Pachymeningitides’

#2 ‘Extrapulmonary tuberculosis’ OR ‘Extra pulmonary tuberculosis’

#3 'meningitis'/exp OR Meningitides OR Pachymeningitis OR Pachymeningitides

#4 'cerebrospinal fluid'/exp OR ‘Cerebrospinal Fluids’ OR ‘Fluid, Cerebrospinal’ OR ‘Fluids, Cerebrospinal’ OR ‘Cerebro Spinal Fluid’ OR ‘Cerebro Spinal Fluids’ OR ‘Fluid, Cerebro Spinal’ OR ‘Fluids, Cerebro Spinal’ OR ‘Spinal Fluid, Cerebro’ OR ‘Spinal Fluids, Cerebro’

#5 #1 OR #2 OR #3 OR #4

#6 'high throughput sequencing'/exp OR 'Metagenomic Next-Generation Sequencing' OR mNGS

#7 #5 AND #6

CNKI AND Wanfang

(结核性脑膜炎 OR 脑膜炎 OR 脑脊液 OR 肺外结核)*主题:(高通量 OR 二代测序 OR 宏基因组)

Pub 53

Embase 507

Cochrane 0

Wanfang 295

Cnki 242
